# Clinicopathological analysis of 124 cardiac tumors: a single-center retrospective study

**DOI:** 10.3389/fcvm.2026.1903934

**Published:** 2026-09-07

**Authors:** Shuai Luo, Xinyi Xu, Die He, Jinjing Wang

**Affiliations:** Department of Pathology, Affiliated Hospital of Zunyi Medical University, Zunyi, Guizhou, China

**Keywords:** cardiac tumors, clinical pathology, diagnosis, differential diagnosis, single center

## Abstract

**Objective:**

To characterize the clinicopathological features, pathological spectrum, and anatomical distribution of cardiac tumors and to evaluate the diagnostic challenges associated with these rare lesions.

**Methods:**

A retrospective analysis was performed on 124 surgically resected cardiac tumors diagnosed at the Affiliated Hospital of Zunyi Medical University between January 2012 and June 2026. Histological examination, immunohistochemistry, and fluorescence *in situ* hybridization (FISH), when indicated, were reviewed. All tumors were reclassified according to the 2021 WHO Classification of Thoracic Tumours. Clinicopathological characteristics and exploratory statistical analyses were performed.

**Results:**

Among the 124 cardiac tumors, myxoma constituted the predominant entity (104/124, 83.9%). A marked predilection for the left atrium was noted, with 78.8% of myxomas located there (82/104), significantly surpassing the frequency in the right atrium (*P* < 0.001). Although malignant tumors were uncommon (12 cases), they exhibited considerable histopathological diversity. Exploratory analysis revealed a higher proportion of malignant tumors in patients aged ≤ 50 years compared to older patients (19.1% vs. 3.9%, *P* = 0.013).

**Conclusions:**

This single-center retrospective series highlights the diverse histopathological spectrum of cardiac tumors, affirming myxoma as the predominant tumor type with a strong left atrial predilection. Although rare, malignant tumors presented diagnostic challenges, necessitating multidisciplinary evaluation that integrates morphology, immunohistochemistry, and molecular testing. The observed age-related difference in the distribution of malignancy should be regarded as exploratory and requires further validation.

## 1 Introduction

Cardiac tumors are clinically rare and can be categorized into primary and secondary types, with primary tumors being even less common. Current international literature reports the incidence of primary cardiac tumors at only 0.0017%–0.19%, with 75% classified as benign and 25% as malignant (1). The clinical diagnosis and pathological differentiation of cardiac tumors present significant challenges. This study retrospectively analyzed 124 cardiac tumor cases diagnosed at our hospital between January 2012 and June 2026. As a retrospective case series, it aims to provide a descriptive overview supported by exploratory statistical observations, emphasizing rare entities, histological diversity, and practical diagnostic considerations, thereby enhancing clinicians' and pathologists' understanding of this uncommon disease group.

## 2 Materials and methods

### 2.1 Clinical data

The study included 124 surgically resected cardiac tumors diagnosed at the Affiliated Hospital of Zunyi Medical University from January 2012 to June 2026. Inclusion criteria were: (1) surgically resected tumors with pathological confirmation; and (2) complete clinical and imaging information. Cases were excluded if obtained solely via needle biopsy or autopsy, or if clinicopathological data were incomplete. Review of all cases was conducted independently by two senior pathologists, with discrepancies resolved through consensus discussion.

### 2.2 Methods

All specimens were fixed in 10% neutral-buffered formalin, routinely paraffin-embedded, sectioned at 3 μm thickness, and stained with hematoxylin and eosin (HE). Immunohistochemical staining utilized the EnVision two-step method, with antibody selection guided by morphological findings and differential diagnostic considerations. This included, but was not limited to, markers of epithelial, mesenchymal, vascular, lymphoid, melanocytic, and neuroendocrine differentiation.

The complete antibody panel comprised vimentin, CK, CK5/6, CK7, CK19, EMA, calretinin, D2-40, TTF1, CD31, CD34, CD117, Factor VIII, ERG, SMA, desmin, MyoD1, myogenin, S100, SOX-10, HMB45, MC, STAT6, MUC4, MDM2, CD68/KP1, H3K27me3, INI-1, LCA, CD20, CD79a, CD3, CD5, CD10, Bcl-6, MUM1, CD43, cyclin D1, Ki-67, NSE, CgA, Syn, CD56, CD99, Bcl-2, P40, p63, CAM5.2, TdT, MPO, CD21, CD23, CD1a, F8, and pan-CK.

All tumors were reclassified according to the 2021 WHO Classification of Thoracic Tumors (5th edition).

### 2.3 Statistical analysis

Statistical analysis was performed using SPSS version 26.0. Categorical variables were expressed as numbers and percentages, with group comparisons conducted using Pearson's *χ*^2^ test or Fisher's exact test as appropriate. A two-sided *P* value < 0.05 was considered statistically significant.

### 2.4 Ethical approval

This study received approval from the Ethics Committee of the Affiliated Hospital of Zunyi Medical University (Approval No.: KELL-2026-036). The requirement for informed consent was waived due to the retrospective nature of the study.

## 3 Results

**Figure 1:**
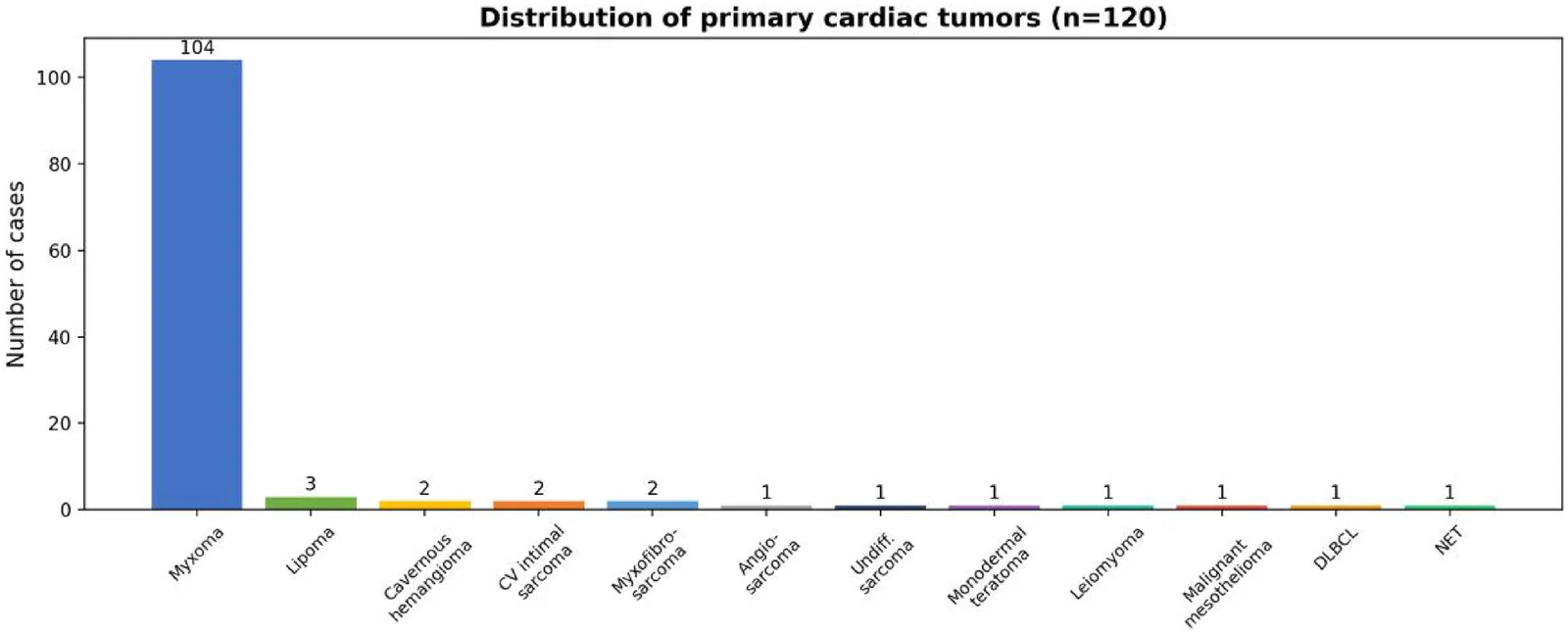
Distribution of primary cardiac tumors (*n* = 120). Bar chart showing the frequency of each tumor type in the surgical cohort.

Among the 124 cardiac tumors (Figure 1), myxoma represented the predominant entity (104/124, 83.9%). Additional tumor types included lipoma (3 cases), cavernous hemangioma (2 cases), monodermal teratoma (1 case), leiomyoma (1 case), cardiovascular intimal sarcoma (2 cases), angiosarcoma (1 case), undifferentiated sarcoma (1 case), myxofibrosarcoma (2 cases), malignant mesothelioma (1 case), diffuse large B-cell lymphoma (1 case), neuroendocrine tumor (1 case), and metastatic tumors (4 cases).

**Figure 2:**
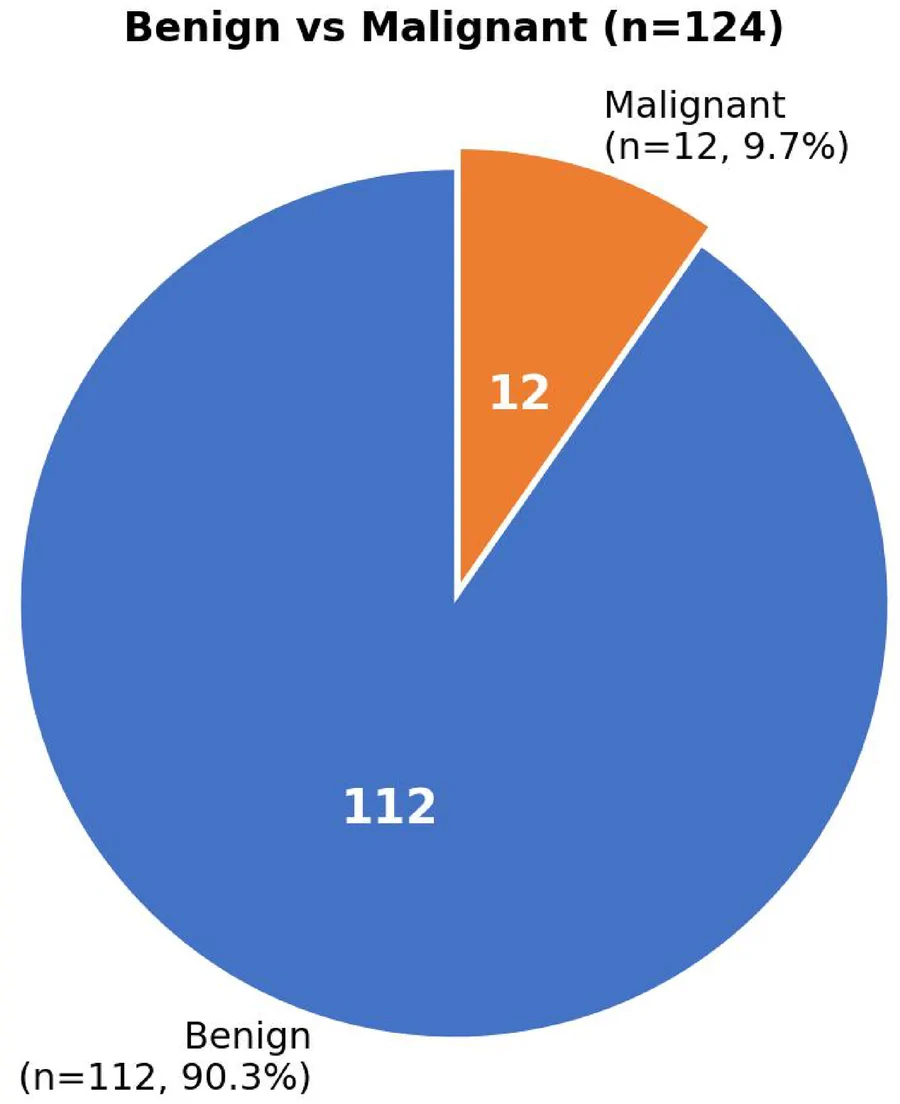
Benign vs malignant tumors in the surgical cohort (*n* = 124). Pie chart showing the overall proportions of benign (*n* = 112, 90.3%) and malignant (*n* = 12, 9.7%) tumors.

**Figure 3:**
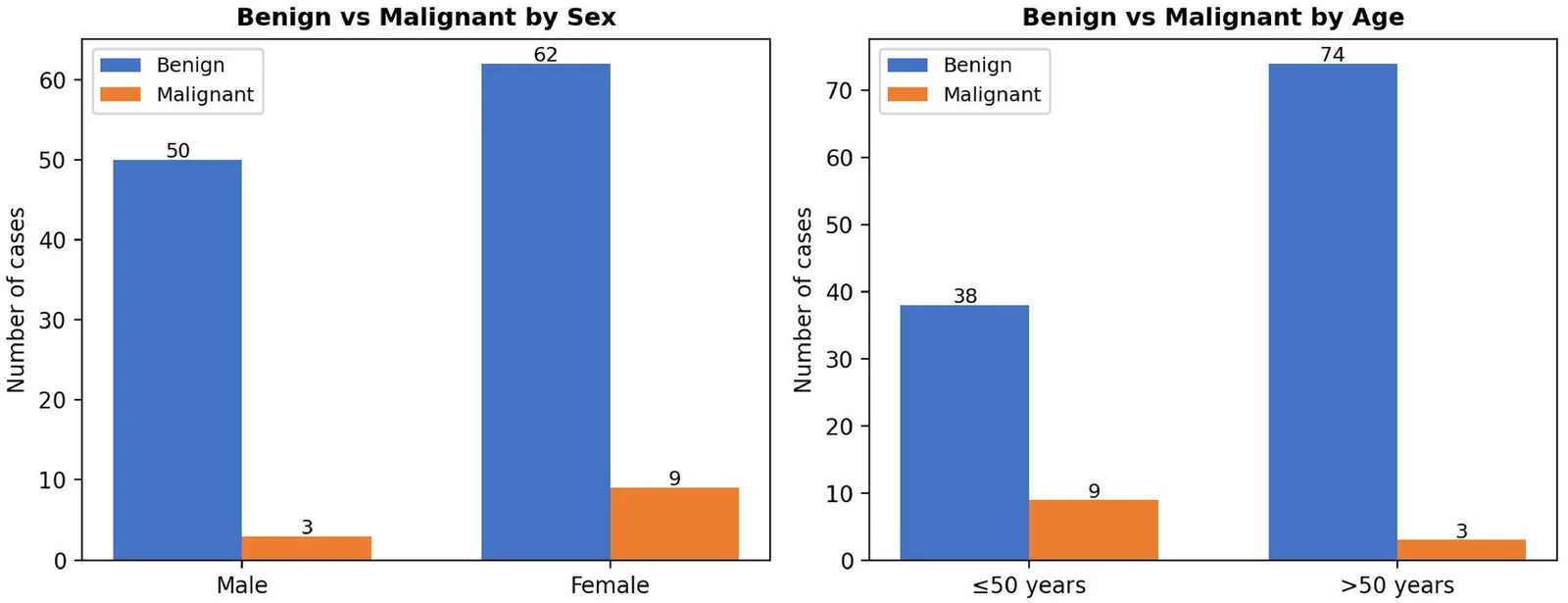
Distribution of benign vs malignant tumors by sex and age.

The distribution of benign and malignant tumors according to sex and age is summarized in Figures 2, 3. Among benign tumors, 50 patients were male and 62 female; malignant tumors occurred in 3 males and 9 females. Regarding age distribution, benign tumors were found in 38 patients aged ≤ 50 years and 74 patients aged > 50 years, while malignant tumors were identified in 9 patients aged ≤ 50 years and 3 patients aged > 50 years.

### 3.1 Myxoma (104 cases)

**Figure 4:**
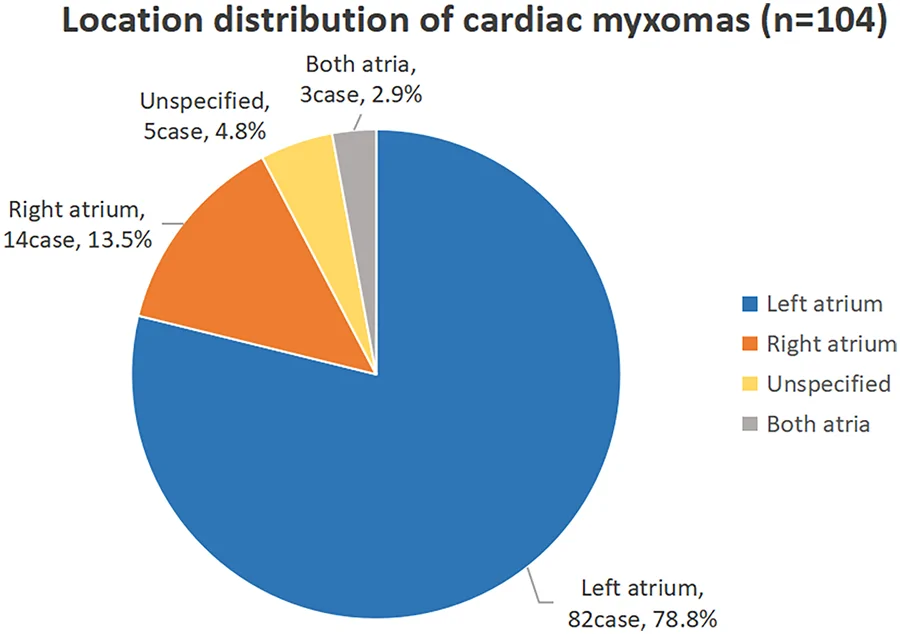
Location distribution of cardiac myxomas (*n* = 104). Pie chart showing the anatomical distribution of myxomas. LA, left atrium; RA, right atrium.

The 104 myxomas comprised 46 males (44.2%) and 58 females (55.8%), with ages ranging from 5 to 78 years. Tumor locations (Figure 4) included 82 cases (78.8%) in the left atrium, 14 cases (13.5%) in the right atrium, both atria in 3 cases (2.9%), and unspecified sites in 5 cases (4.8%). The incidence of left atrial involvement was significantly higher than that of right atrial involvement (*χ*^2^ = 52.128, *P* < 0.001). Clinical manifestations included circulatory symptoms (chest tightness, dyspnea, palpitations, and chest pain), respiratory symptoms (cough and dyspnea), and neurological symptoms (dizziness and syncope).

**Figure 5:**
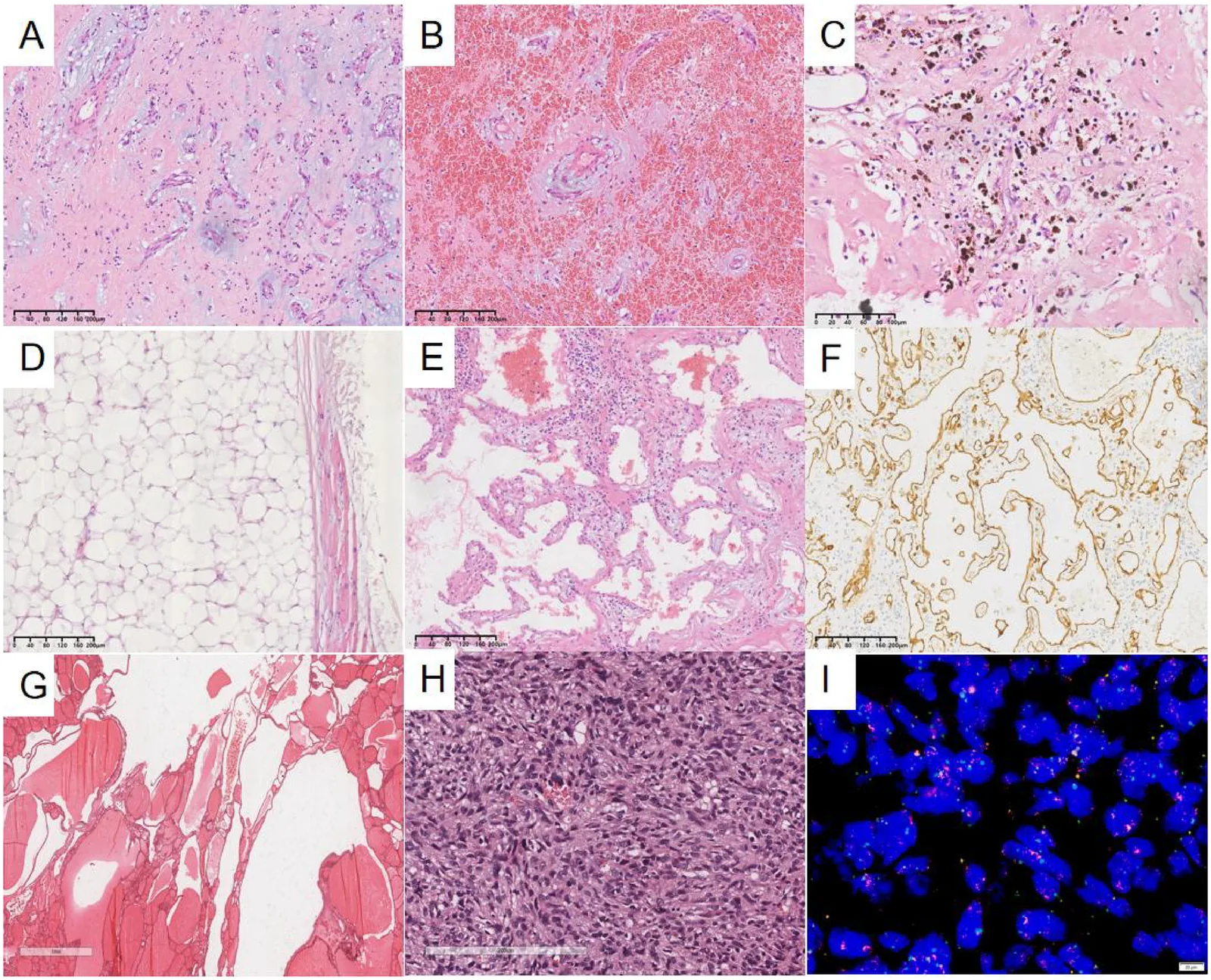
Histological features of cardiac tumors. Under microscopy, small nest-like or solitary scattered tumor cells are observed within the mucinous stroma of a myxoma **(A)**; a typical “myxoid halo ring” with hemorrhage is visible **(B)**; cases accompanied by hemosiderin deposition **(C)**, lipoma **(D)**, and cavernous hemangioma **(E)**; in cavernous hemangioma, CD34 staining reveals positive vascular walls **(F)**; simple goiter **(G)**; cardiac intimal sarcoma **(H)**; and cardiac intimal sarcoma showing MDM2 amplification **(I)**.

Histologically (Figures 5A–C), myxomas displayed scattered spindle and stellate cells within a myxoid stroma, with rare mitotic figures. In cases with heightened mitotic activity, tumor cells clustered around vessels, forming rosette-like structures. A characteristic myxoid halo appeared as pale blue material surrounding vascular endothelial cells against an eosinophilic background. Five cases were associated with thrombus formation. Immunohistochemical analysis in 10 cases demonstrated positivity for vimentin, calretinin, CD31, and CD34, while being negative for desmin.

### 3.2 Lipoma (3 cases)

**Figure 6:**
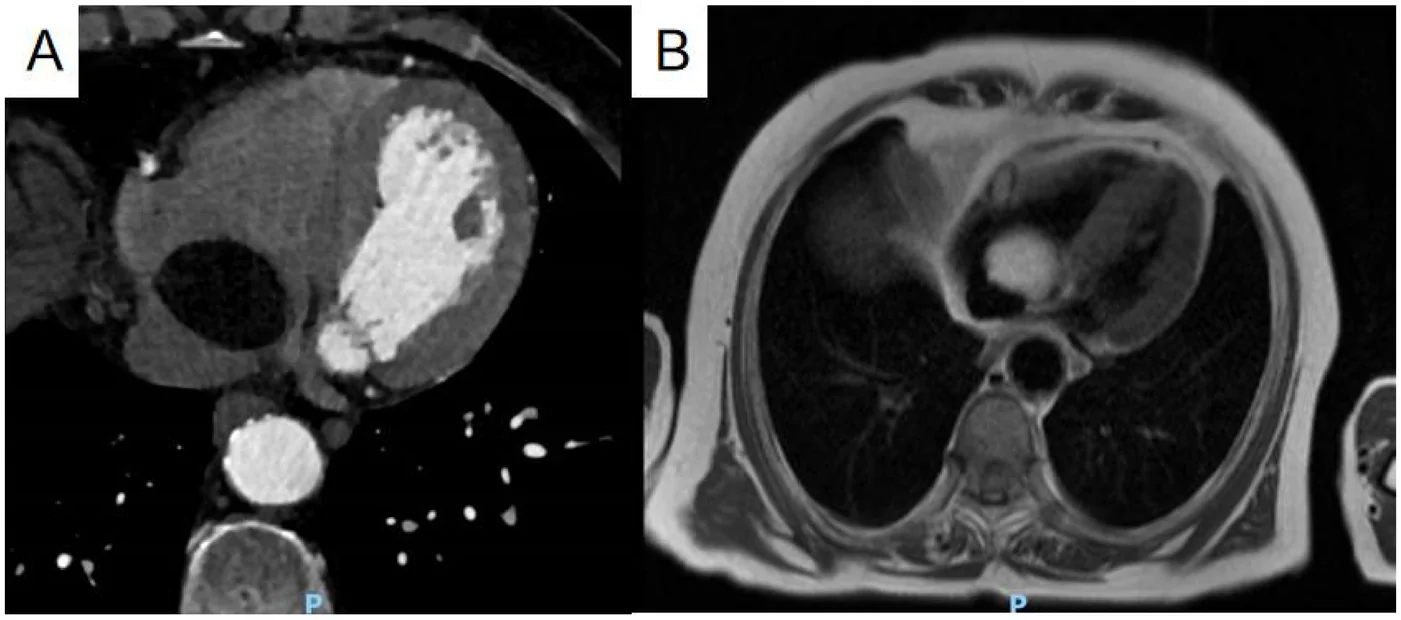
Imaging features of cardiac lipoma. CT imaging of cardiac lipoma shows a hypodense lesion **(A)**, while MRI reveals a fat signal **(B)**.

Three lipomas were identified, consisting of two males (aged 51 and 75 years) and one female (44 years), with a mean age of 57 years. All lesions were located in the right atrium, measuring 2–4.5 cm. Coronary CT angiography (Figure 6A) revealed fatty masses adjacent to the inferior margin of the right atrium. Cardiac MRI (Figure 6B) showed nodular lesions on the right atrial surface of the atrial septum, characterized by short T1 and long T2 signals.

Grossly, the tumors were encapsulated, with gray-yellow soft cut surfaces. Histologically (Figure 5D), they consisted of mature adipose tissue encased by a fibrous capsule. The adipocytes displayed clear lipid-rich cytoplasm without atypia, and mitotic figures were rare. All cases were diagnosed as cardiac lipoma.

### 3.3 Angiocavernoma (2 cases)

The two cases of cavernous hemangioma involved the left ventricle and left atrial appendage in a 23-year-old male and a 52-year-old female, respectively. The tumors measured 25 × 20 × 5 mm and 75 × 55 × 40 mm. Grossly, the lesions appeared gray-white, with one exhibiting a central gray-brown area, while the other was encapsulated with a cystic-solid cut surface containing hemorrhagic fluid. Histologically (Figure 5E), both tumors displayed irregularly dilated vascular spaces with indistinct borders between the lesions and adjacent myocardium. Vascular lining cells tested positive for vimentin, CD34 (Figure 5F), and Factor VIII while being negative for pan-CK, D2-40, HMB45, and S100. Smooth muscle actin (SMA) and desmin highlighted smooth muscle components in the walls of some proliferating vessels.

### 3.4 Cardiac simple goiter (1 case)

A 51-year-old male presented with a right ventricular tumor measuring 65 × 45 × 25 mm. The lesion was encapsulated with clear borders, displaying a gray-red, soft, partially gelatinous cut surface with hemorrhage and calcification.

Histologically (Figure 5G), the tumor was predominantly composed of thyroid tissue with follicles of varying sizes containing eosinophilic colloid. The follicles were lined by bland cuboidal or low columnar epithelial cells, with occasional oncocytic, clear, and mucinous cells noted, along with focal hemorrhage and cystic degeneration.

### 3.5 Leiomyoma (1 case)

A 49-year-old female presented with a tumor involving the right atrium and inferior vena cava. Histologically, the lesion consisted of spindle cells arranged in fascicles and a storiform pattern, characterized by eosinophilic cytoplasm and mild atypia. No mitotic figures were identified (0/50 HPF). Immunohistochemistry revealed positivity for desmin, H-caldesmon, and SMA, while being negative for S100, with a Ki-67 proliferation index of < 5%. Despite increased cellularity, the low proliferative activity and absence of atypical features supported a diagnosis of benign leiomyoma. Clinical evaluation showed no evidence of uterine leiomyoma or intravenous leiomyomatosis with cardiac extension, leading to a final diagnosis of primary cardiac leiomyoma.

### 3.6 Cardiovascular intimal sarcoma (2 cases)

Both cases of cardiovascular intimal sarcoma occurred in the left atrium, involving male patients aged 49 and 55 years. The tumors measured 40 × 40 × 10 mm and 30 × 25 × 7 mm, respectively. Histologically (Figure 5H), both lesions consisted of spindle or short spindle cells within a myxoid background, accompanied by moderate atypia and pathological mitotic figures. Immunohistochemical analysis indicated expression of vimentin and SMA in both cases. Case 1 exhibited focal positivity for desmin and SATB2, with negativity for CK, EMA, CD34, and MDM2, and a Ki-67 index of 15%. Case 2 demonstrated positivity for MDM2 and focal ERG expression, while being negative for EMA, desmin, CD31, CD34, and S100, with a Ki-67 index of approximately 40%. FISH confirmed MDM2 gene amplification in both cases (Figure 5I), with notable discordance observed in Case 1, where negative immunohistochemical staining contrasted with positive FISH amplification, suggesting that negative MDM2 protein expression does not exclude MDM2 gene amplification.

### 3.7 Angiosarcoma (1 case)

**Figure 7:**
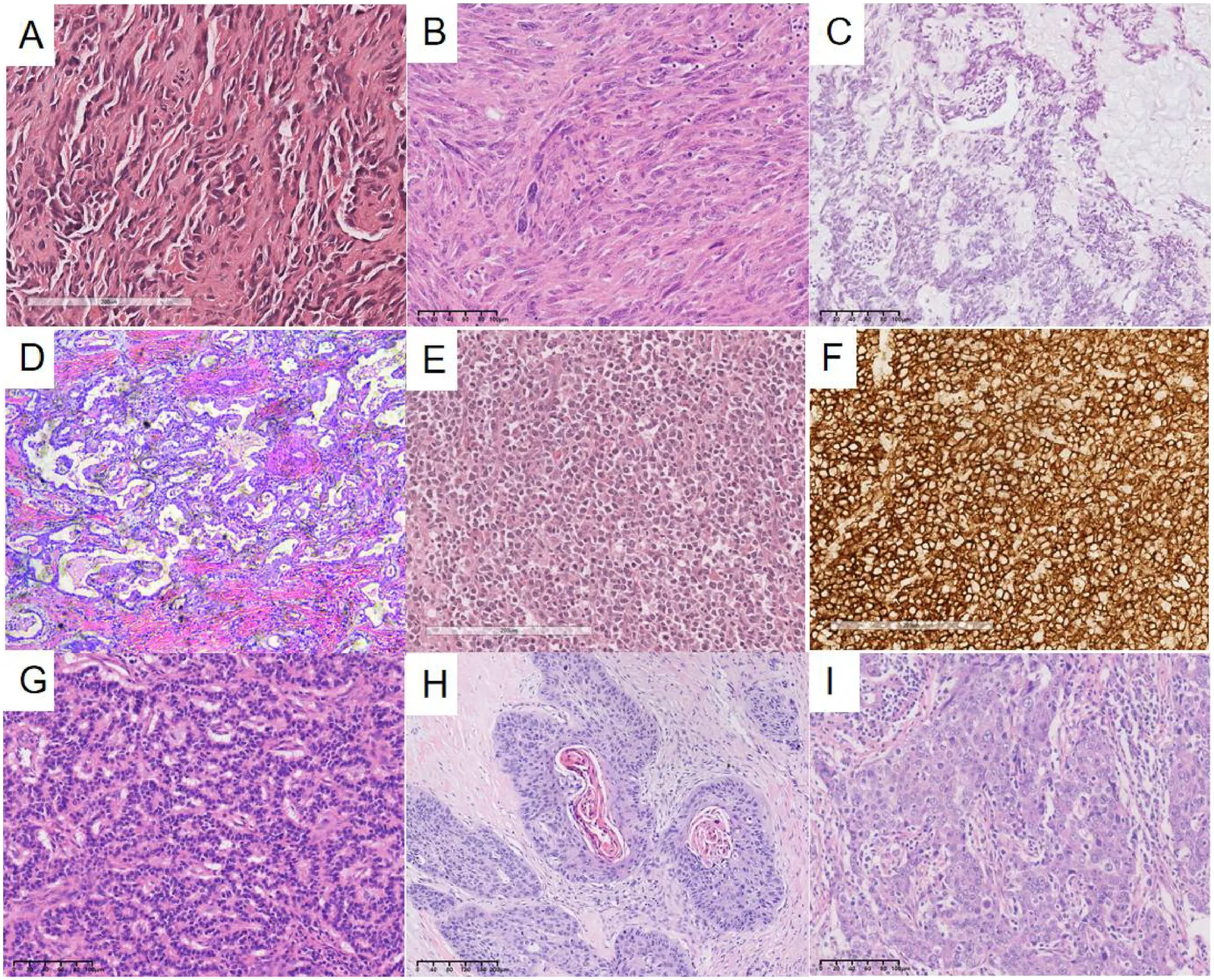
Microscopic images showing angiosarcoma **(A)**, undifferentiated sarcoma **(B)**, myxofibrosarcoma **(C)**, mesothelioma **(D)**, diffuse large B-cell lymphoma **(E)**, CD20 strongly diffusely positive diffuse large B-cell lymphoma **(F)**, neuroendocrine tumor **(G)**, metastatic keratinizing squamous cell carcinoma **(H)**, and metastatic non-keratinizing squamous cell carcinoma **(I)**.

A 29-year-old female presented with a right atrial tumor measuring 6 × 5 × 2 mm. Histologically (Figure 7A), the tumor displayed features resembling cardiac endocardial sarcoma. Immunohistochemistry revealed diffuse positivity for ERG, CD31, and CD34, alongside additional positivity for SMA and vimentin. The Ki-67 index was approximately 40%. The tumor was negative for MDM2 amplification by FISH and lacked expression of epithelial, mesothelial, and other lineage markers.

### 3.8 Undifferentiated sarcoma (1 case)

A 22-year-old female presented with a left atrial tumor measuring 15–30 mm. The lesion lacked a capsule and displayed a gray-white to gray-brown, moderately firm cut surface. Histologically (Figure 7B), the tumor comprised highly atypical spindle and epithelioid cells with giant cells and frequent mitotic figures, leading to a final diagnosis of undifferentiated sarcoma. Immunohistochemistry revealed positivity for vimentin and CD68 (KP1), focal positivity for desmin and SMA, and negativity for epithelial, vascular, melanocytic, and neural markers, with a Ki-67 index of approximately 30%.

### 3.9 Myxofibrosarcoma (2 cases)

Two cases of myxofibrosarcoma were identified, both in female patients with left atrial involvement. The tumors measured 60 × 60 × 20 mm, with one case affecting the mitral valve region. Histologically (Figure 7C), both lesions comprised atypical spindle cells arranged in storiform or fascicular patterns within a myxoid stroma, exhibiting increased cellularity, nuclear atypia, and significant mitotic activity. Immunohistochemistry in Case 1 showed positivity for vimentin and focal SMA expression, while CK, EMA, S100, desmin, MyoD1, CD31, CD34, STAT6, MDM2, and MUC4 were negative. Retention of H3K27me3 and INI-1 expression was noted, with a Ki-67 index of approximately 30% in hot spots. Based on overlapping morphological features and immunophenotypic profiles, both tumors were diagnosed as left atrial myxofibrosarcoma.

### 3.10 Malignant mesothelioma (1 case)

An 18-year-old female presented with a left atrial tumor measuring 50 mm in diameter. Grossly, the tumor showed a papillary surface with a gray-white to gray-brown soft cut surface. Histologically (Figure 7D), the tumor exhibited myxoid stromal changes accompanied by tubular and microcystic structures resembling an adenomatoid tumor. The tumor cells appeared vacuolated and cuboidal, with indications of stromal invasion. Immunohistochemistry demonstrated positivity for calretinin, CK, EMA, and vimentin while being negative for CK5/6 and TTF1. The final diagnosis was epithelioid malignant mesothelioma of the left atrium.

### 3.11 Diffuse large B-cell lymphoma (1 case)

A 42-year-old female presented with a right atrial mass. Gross examination revealed fragmented gray-white to gray-brown tissue measuring 7.0 × 6.8 × 3 cm, which was partially encapsulated, with a soft cut surface and relatively clear borders. Histologically (Figure 7E), the tumor exhibited diffuse proliferation of large lymphoid cells infiltrating the myocardium. The cells showed vesicular nuclei, prominent nucleoli, basophilic to amphophilic cytoplasm, frequent mitotic figures, apoptosis, and focal necrosis.

Immunohistochemistry demonstrated positivity for LCA, CD20 (Figure 7F), CD79a, MUM1, and CD43, alongside partial Bcl-6 expression, while being negative for CD10, CD3, CD45RO, CD5, cyclin D1, CK, EMA, and MPO. The Ki-67 index was approximately 70%. Based on clinical, morphological, and immunophenotypic findings, the tumor was diagnosed as primary cardiac diffuse large B-cell lymphoma (PC-DLBCL; non-germinal center subtype).

### 3.12 Neuroendocrine tumor (1 case)

A 51-year-old female presented with a left ventricular tumor measuring 3 × 2 × 1.5 mm. Grossly, the lesion was a well-circumscribed, encapsulated gray-yellow mass with a moderately firm cut surface. Histologically (Figure 7G), the tumor demonstrated an organoid growth pattern composed of nests, cords, ribbons, trabeculae, and rosette-like structures. Tumor cells were relatively uniform, featuring round to polygonal nuclei, fine granular chromatin, eosinophilic or clear cytoplasm, and rare mitotic figures. These tumor cells were separated by abundant sinusoidal vessels and minimal fibrous stroma.

Immunohistochemistry showed positivity for CK, CD56, CgA, Syn, MyoD1 (membranous), and NSE, while being negative for S100, desmin, SMA, and vimentin, with a Ki-67 index of 2%. Combined with clinical history and imaging findings showing no extracardiac primary lesion, the tumor was diagnosed as a primary cardiac neuroendocrine tumor (G1).

### 3.13 Metastatic tumors (4 cases)

Four metastatic cardiac tumors were identified, originating from lung squamous cell carcinoma, nasopharyngeal squamous cell carcinoma, lung adenocarcinoma, and thymic squamous cell carcinoma. The metastatic lesions involved the atrium, left atrial wall, or left ventricle. One case of lung squamous cell carcinoma (Figure 7H) affected atrial tissue in a 64-year-old male. A left atrial metastatic squamous cell carcinoma (Figure 7I) in a 52-year-old female involved the right superior pulmonary vein and displayed expression of CK, CK5/6, P40, and EBER/ISH, supporting a nasopharyngeal origin. Another case involved left atrial and adjacent bronchial infiltration by lung adenocarcinoma in a 42-year-old female, showing immunoreactivity for CK, TTF1, and desmin. The fourth case involved thymic squamous cell carcinoma invading the left ventricle in a 46-year-old female, exhibiting positivity for pan-CK, CD117, CD5, CK5/6, and p63, consistent with thymic origin.

### 3.13.1 Exploratory statistical observations

Exploratory statistical observations

Exploratory statistical analysis was performed on the 124 cardiac tumors, comprising 120 primary cardiac tumors (Table 1) and 4 metastatic tumors (Table 2). Correlation analysis (Table 3) revealed no significant difference in sex distribution between benign and malignant tumors (benign: 50 males and 62 females; malignant: 3 males and 9 females; Fisher's exact test, *P* = 0.232). Age-stratified analysis indicated that malignant tumors were more frequent in patients aged ≤ 50 years compared to those aged > 50 years (9/47, 19.1% vs. 3/77, 3.9%; *χ*^2^ = 6.107, *P* = 0.013). No significant difference was identified in sex distribution between myxoma and non-myxoma tumors (myxoma: 46 males and 58 females; non-myxoma: 7 males and 12 females; *χ*^2^ = 0.098, *P* = 0.754). In contrast, myxomas exhibited a strong left atrial predilection, with significantly more cases affecting the left atrium than the right atrium (82 vs. 14 cases; *χ*^2^ = 52.128, *P* < 0.001).

**Table 1:** Clinicopathological analysis of 120 primary cardiac tumors.

No.	Tumor type	*n*	Age (years)	M:F	Tumor diameter (cm)	Location
1	Myxoma	104	51 (5–78)	46:58	0.5–8.5	LA (82), RA (14), Bi (3)
2	Lipoma	3	56 (44–75)	2:1	2–4.5	RA (3)
3	Cavernous hemangioma	2	23–52	1:1	2.5–7.5	LV (1), LA (1)
4	Cardiovascular intimal sarcoma	2	49–55	2:0	3.0–4.0	LA (2)
5	Myxofibrosarcoma	2	18–38	0:2	3–6	LA (2)
6	Monodermal teratoma	1	51	1:0	6.5	RV (1)
7	Leiomyoma	1	49	0:1	4.2	RA (1)
8	Angiosarcoma	1	29	0:1	0.6	RA (1)
9	Undifferentiated sarcoma	1	22	0:1	3	LA (1)
10	Malignant mesothelioma	1	18	0:1	5	LA (1)
11	Diffuse large B-cell lymphoma	1	42	0:1	4.3	RA (1)
12	Neuroendocrine tumor	1	51	0:1	2.8	LV (1)

**Table 2:** Clinicopathological analysis of 4 secondary cardiac tumors.

No.	Tumor type	Primary site	*n*	Age (years)	Sex	Location
1	Keratinizing SCC	Lung	1	64	M	LA
2	Non-keratinizing SCC	Nasopharynx	1	52	F	LA
3	Non-keratinizing SCC	Anterior mediastinum	1	46	F	LV
4	Adenocarcinoma (solid type)	Lung	1	42	F	LA

**Table 3:** Chi-square test analysis of clinicopathological features.

Comparison	Group	*n*	*χ*²	*P* value
Benign/malignant×sex	Benign	50 (M)/62 (F)	1.000¹	0.232
	Malignant	3 (M)/9 (F)		
Benign/malignant×age	Benign (≤50 yrs)	38	6.107	0.013
	Benign (>50 yrs)	74		
	Malignant (≤50 yrs)	9		
	Malignant (>50 yrs)	3		
Myxoma×sex	Myxoma	46 (M)/58 (F)	0.098	0.754
	Non-myxoma	7 (M)/13 (F)		
Myxoma location	Left atrium	82	52.128	<0.001
	Right atrium	14		

## 4 Discussion

### 4.1 Myxoma

In this series, 104 myxomas accounted for 86.7% of all primary tumors, establishing myxoma as the predominant tumor type in the cohort. This proportion was slightly higher than the 75%–80% reported in previous studies, potentially reflecting referral bias due to the hospital's status as a regional cardiac center. Anatomically, the left atrium was significantly more frequently involved (82 cases, 78.8%) than the right atrium (14 cases, 13.5%) (*χ*^2^ = 52.128, *P* < 0.001), consistent with the recognized predilection of myxomas for the left atrium (2, 3). The male-to-female ratio was 46:58, without significant difference (*P* = 0.754), which does not entirely align with the female predominance observed in some studies, possibly reflecting differences in population characteristics and sample size.

All five patients with thrombus formation presented with embolic symptoms, indicating that the thromboembolic risk requires particular attention in myxoma patients with associated thrombosis. Histologically, myxomas exhibited typical hypocellular myxoid stroma with scattered stellate cells. The absence of significant nuclear atypia and mitotic activity differentiates myxomas from myxoid sarcomas. The characteristic myxoid halo surrounding blood vessels remains a key diagnostic clue. In five cases, the tumor attachment site was not clearly documented, highlighting the need for improved anatomical descriptions in clinical records.

### 4.2 Lipoma

All three lipomas in this series were located in the right atrium and were incidentally detected through imaging. Histologically (4, 5), these lesions consisted of mature adipocytes without nuclear atypia or lipoblasts, supporting a benign diagnosis. In this cohort, imaging findings combined with characteristic morphology were sufficient for diagnosis, suggesting that surveillance may be appropriate for asymptomatic cardiac lipomas.

### 4.3 Cavernous hemangioma

The two cavernous hemangioma cases exhibited different clinical presentations despite similar benign pathological characteristics. One left ventricular lesion caused chest pain, highlighting that symptoms can occur even in small tumors. Histologically, cavernous hemangiomas consisted of dilated blood-filled vascular channels lined by bland endothelial cells, with immunohistochemical findings indicating vascular differentiation. The characteristic vascular phenotype (CD31+, CD34+, Factor VIII+, D2-40−) and absence of malignant features were consistent with previous reports (6, 7).

### 4.4 Monodermal teratoma

The case involving a 51-year-old male with a right ventricular tumor was pathologically confirmed as monodermal teratoma (struma cordis). Cardiac teratomas, particularly the thyroid-predominant monodermal subtype, are extremely uncommon (8, 9). Diagnosis relied on identifying mature thyroid follicles and excluding metastatic thyroid carcinoma through clinical examination and immunohistochemical evaluation.

### 4.5 Leiomyoma

The single cardiac leiomyoma case occurred in a 49-year-old woman, involving the right atrium and inferior vena cava. The primary diagnostic challenge lay in distinguishing primary cardiac leiomyoma from intravenous leiomyomatosis and benign metastasizing leiomyoma (10, 11). In this patient, the absence of uterine lesions and pelvic venous involvement supported a primary cardiac origin. The spindle cell morphology, absence of mitotic activity (0/50 HPF), and low Ki-67 index further indicated benign behavior. This case highlights the importance of integrating clinical history, imaging findings, and pathological examination when evaluating smooth muscle tumors involving the heart.

### 4.6 Cardiovascular intimal sarcoma

Both cases of cardiovascular intimal sarcoma in this cohort involved male patients and affected the left atrium, with MDM2 amplification confirmed by FISH, consistent with prior reports (12). One case exhibited discordance between MDM2 immunohistochemistry and FISH results. Although MDM2 IHC is valuable, its sensitivity is limited, and some cases with amplification may yield negative staining (13). Consequently, FISH analysis remains critical when morphology and clinical findings suggest intimal sarcoma. The overlap between intimal sarcoma and undifferentiated sarcoma highlights the necessity of integrating morphological, immunohistochemical, and molecular findings (14).

### 4.7 Angiosarcoma

The single angiosarcoma case involved a 29-year-old woman with a right atrial tumor. Diffuse expression of ERG, CD31, and CD34, coupled with the absence of MDM2 amplification, supported the diagnosis (15, 16). The primary diagnostic challenge was differentiating this from other malignant cardiac tumors with overlapping endothelial marker expression. A combination of endothelial immunomarkers and molecular findings was essential for accurate classification.

### 4.8 Undifferentiated sarcoma

One case of undifferentiated sarcoma occurred in a 22-year-old woman with a left atrial tumor. Diagnosis was established after excluding other sarcoma subtypes through extensive immunohistochemical evaluation (17, 18). The tumor exhibited marked pleomorphism, frequent mitotic activity, and no specific line of differentiation. Previously reported overlapping molecular characteristics between undifferentiated sarcoma and intimal sarcoma may suggest biological similarities between these entities (14).

### 4.9 Myxofibrosarcoma

Both myxofibrosarcoma cases in this series occurred in female patients and involved the left atrium. One case required differentiation from myxoma due to overlapping myxoid features; however, increased cellularity, cytological atypia, mitotic activity, and elevated Ki-67 expression supported a malignant diagnosis (19, 20). The second case displayed high-grade sarcomatous features involving the mitral valve. These cases highlight the importance of evaluating cellularity, atypia, proliferation activity, and myxoid components when distinguishing myxofibrosarcoma from benign myxoid tumors.

### 4.10 Malignant mesothelioma

The primary pericardial malignant mesothelioma case in this series involved an 18-year-old patient, considerably younger than the commonly reported age range (21–23). Diagnosis was established based on characteristic morphology and immunohistochemical findings, along with exclusion of metastatic adenocarcinoma. This case emphasizes that malignant mesothelioma should be considered in the differential diagnosis of pericardial tumors, even in younger patients.

### 4.11 Diffuse large B-cell lymphoma

The single case of PC-DLBCL occurred in a 42-year-old woman and involved the right atrium. Initially suspected to be myxoma, the final diagnosis was confirmed by pathological examination. Consistent with previous reports, the right atrium is a common site for PC-DLBCL (24, 25). Unlike most primary cardiac tumors, PC-DLBCL requires systemic chemotherapy rather than surgical resection as the main treatment strategy (26).

### 4.12 Neuroendocrine tumor

The single case of primary cardiac neuroendocrine tumor occurred in the left ventricle and was classified as G1 (Ki-67 2%). Diagnosis necessitated the exclusion of metastatic neuroendocrine tumors from other primary sites (27). Whole-body imaging confirmed the absence of extracardiac lesions. This case highlights the importance of comprehensive clinical evaluation when diagnosing rare cardiac neuroendocrine neoplasms.

### 4.13 Metastatic tumors

Four metastatic tumors were identified in this series, originating from lung squamous cell carcinoma, nasopharyngeal squamous cell carcinoma, lung adenocarcinoma, and thymic squamous cell carcinoma. Notably, one metastatic squamous cell carcinoma was initially misdiagnosed as a primary cardiac myxoma before postoperative pathological examination established the correct diagnosis. This finding indicates the diagnostic challenge of distinguishing metastatic lesions from primary cardiac tumors. Previous studies have indicated that a proportion of intracardiac masses initially considered primary tumors may actually represent metastatic disease (27, 28). Thus, integration of clinical history, imaging findings, and comprehensive pathological evaluation is essential during diagnosis. Cardiac metastases are likely under-recognized clinically, as autopsy studies have shown a higher incidence than that detected through routine clinical evaluation (29, 30).

### 4.14 Integrated observations across tumor types

Several findings from this cohort provide broader insights into the clinicopathological characteristics of cardiac tumors. First, benign tumors, particularly myxomas, constituted the majority of cases, whereas malignant tumors were uncommon but exhibited substantial histological heterogeneity. Second, accurate classification of malignant cardiac tumors frequently necessitated integration of histological morphology, immunohistochemistry, and molecular testing, especially for entities with overlapping features. Third, younger patients demonstrated a higher proportion of malignant tumors in this cohort; however, this observation should be viewed as exploratory due to the limited number of malignant cases and requires validation in larger studies. Finally, the identification of metastatic tumors highlights the importance of considering secondary cardiac involvement when evaluating intracardiac masses.

### 4.15 Limitations

This study has several limitations. First, its retrospective, single-center design may have introduced selection bias, and the inclusion of only surgically resected cases limits the generalizability of the findings to the overall population of patients with cardiac tumors. Second, the absence of follow-up data precluded evaluation of long-term outcomes, including survival and tumor recurrence.

## 5 Conclusion

In this single-center retrospective clinicopathological series, cardiac myxoma emerged as the predominant primary cardiac tumor, showing a marked predilection for the left atrium, while malignant cardiac tumors were uncommon yet demonstrated considerable histological diversity. Malignant tumors appeared relatively more frequent among patients ≤ 50 years; however, this observation should be considered exploratory given the limited number of malignant cases and necessitates validation in larger studies. Accurate diagnosis of cardiac masses depends on the integration of clinical information, imaging findings, histomorphology, immunohistochemistry, and molecular testing when appropriate, particularly MDM2 FISH analysis in suspected cardiovascular intimal sarcoma. Despite the limitations of the retrospective design and sample size, the findings contribute to the clinicopathological spectrum of cardiac tumors and highlight the value of a multidisciplinary diagnostic approach.

## Data Availability

The original contributions presented in the study are included in the article/Supplementary Material, further inquiries can be directed to the corresponding authors.
